# Exploring the Relationship Between Stability and Dynamics in Polymer-Based Amorphous Solid Dispersions for Pharmaceutical Applications

**DOI:** 10.3390/polym17091210

**Published:** 2025-04-28

**Authors:** Emeline Dudognon, Jeanne-Annick Bama, Frédéric Affouard

**Affiliations:** Univ. Lille, CNRS, INRAE, Centrale Lille, UMR 8207-UMET-Unité Matériaux et Transformations, F-59000 Lille, France; jeanneannickb@gmail.com (J.-A.B.); frederic.affouard@univ-lille.fr (F.A.)

**Keywords:** amorphous solid dispersions, glass transition, solubility, molecular dynamics, Polyvinylpyrrolidone, Terfenadine

## Abstract

Mixing polymeric excipients with drugs in amorphous solid dispersions (ASD) is known to enhance the bioavailability of drugs by inhibiting their recrystallisation. However, the mechanisms underlying stabilisation remain not fully understood. This study aims to improve our understanding of the role of dynamics, particularly the molecular movements that drive instabilities, through investigations of ASD made of Polyvinylpyrrolidone (PVP K12) and a model drug, Terfenadine. The analyses combine temperature modulated differential scanning calorimetry (MDSC) and dielectric relaxation spectroscopy. The results reveal that the produced ASDs are supersaturated with Terfenadine, regardless of the content, and that PVP slows down the dynamics of the blends, limiting the recrystallisation of the drug during heating. Although the ASDs appear homogeneous based on thermal analysis with a single glass transition consistently detected by MDSC, the investigation of the dynamics reveals a dissociation of the main relaxation into two components for PVP contents below 30 wt.%. This dynamic heterogeneity suggests a structural heterogeneity with the coexistence of two amorphous phases of different compositions, each characterised by its own dynamics. The complex evolution of these dynamics under recrystallisation is rationalised by the confrontation with the phase and state diagram of Terfenadine/PVP blends established by MDSC.

## 1. Introduction

For many decades, one of the major challenges for the pharmaceutical industry has been the poor aqueous solubility of drugs on the market or in the development pipeline [1,2]. The formulation of a solid dispersion of the drug in an excipient appeared as a relevant strategy for oral delivery to enhance the dissolution rate. It has grown even more relevant from the 1960s onwards with the use of polymers as charge carriers, such as Polyvinylpyrrolidone (PVP) [3,4]. Indeed, amorphous polymers such as PVP, Poly(vinylpyrrolidone-co-vinyl acetate) (PVPVA), hydroxypropyl methylcellulose (HPMC) or HMPC acetate succinate (HPMCAS), to cite only the most used, enable the formation of amorphous solid dispersions (ASDs). In these ASDs, the role of the polymeric matrix is to benefit from the enhanced bioavailability of the drug’s amorphous form resulting from its Gibbs free energy, which is higher than the crystalline form, while avoiding its inherent instability by preventing its re-crystallisation during storage. The polymeric matrix also enables controlling the drug release upon dissolution and delaying the re-precipitation prolonging the supersaturation of the solution relative to the solubility of the crystalline form (parachute effect [5]) [6,7]. Therefore, ASDs have received increasing interest for several years. However, despite numerous research works in recent years, the mechanisms of drug stabilisation by the polymeric matrix are not fully understood and it remains a challenge to predict the most suitable excipient to use [3].

A thermodynamic and a kinetic factor are involved. On the one hand, the solubility of the drug in the polymer has to be assessed to avoid supersaturation that could lead to re-crystallisation of the excess drug. This solubility strongly depends on the nature of the polymer and on the interactions developing between the polymer and the drug [8]. On the other hand, the stabilisation of the drug by the polymer implies the miscibility of both compounds. The generally accepted sign of mixing at the molecular scale of two amorphous compounds having different glass transitions is the detection of a single glass transition for the mixture. The determination of the glass transition temperature *T_g_* of the blend, the temperature at which wide amplitude motions are frozen, also reflects the kinetic factor, which has a key role in stability. Indeed, to occur, crystallisation (nucleation and growth) requires the mobility of the molecules. A reduction in mobility, therefore, favours a reduction in the probability of crystallisation, and an increase of the ASD *T_g_* (antiplasticising effect) through mixing with a high *T_g_* polymer is an important factor preventing crystallisation [3]. The physical stability of an ASD is, thus, often addressed by determining the phase and state diagram showing the evolutions of the drug solubility and the *T_g_* of the blend as a function of the ASD composition. It allows to identify the different domains: mono or biphasic, crystalline or amorphous, and stable or unstable. However, considering the effects of mobility via *T_g_* variations is a limited approach. Further research works dedicated to the characterisation of the ASD’s dynamics by specific techniques, such as dielectric relaxation spectroscopy (DRS), were carried out in recent years in order to more precisely identify the parameters that reduce the mobility and hamper the re-crystallisation (nucleation) of the drug. These include interactions developed between the polymer and the drug [9,10,11], the polymer concentration [12] or the polymer macromolecular architecture parameters like the molecular weight [13], the chain length [14], and the polymer enantiomerism [15]. It is, however, also crucial to understand the molecular movements at the origin of instabilities that could lead to demixing phenomena and re-crystallisation of the drug.

In the presented work, we report a careful investigation of a model ASD composed of Terfenadine and PVP K12. Terfenadine is a hydrophobic molecule with a poor aqueous solubility (10.2 µg.mL^−1^) [16] that can be easily amorphised by quench from the melt or by milling at room temperature (*T_g_* = 60 °C). Its molecular mobility has been previously investigated by DRS experiments and molecular dynamics simulations [17]. PVP is a biocompatible and non-toxic polymer widely used in the pharmaceutical industry [18]. It is a linear polymer constituted of an apolar backbone with pendant polar rings (pyrrolidone moieties), and it can interact with both hydrophilic and lipophilic drugs. PVP is an amorphous polymer and the glass transition temperature of PVP K12 (pharmaceutical grade) is close to 100 °C. The main objective of this work is to explore the relationship between the stability and dynamics of TFD/PVP ASDs through the characterisation of ASDs with different TFD contents by temperature modulated differential scanning calorimetry (MDSC) and DRS. On the one hand, the investigation by MDSC enables us to determine the phase and state diagram of TFD/PVP blends; on the other hand, the investigation by DRS gives us insight into their dynamics revealing structural heterogeneities. Their evolution under re-crystallisation is then rationalised by the confrontation with the phase and state diagram.

## 2. Materials and Methods

### 2.1. Materials

Terfenadine (α-[4-(1,1-Dimethylethyl)phenyl]-4-(hydroxydiphenylmethyl)-1-piperidinebutanol, C_32_H_41_NO_2_, CAS 50679-08-8, M = 471.67 g.mol^−1^) was supplied by Sigma Aldrich (purity ≥ 97.5%, Lt MKBX6318V) and was used as received. It is a chiral molecule and the studied compound contains the same amount of each enantiomers (specific rotation angle of 0°, c = 1 in chloroform, 298 K). PVP K12 (M_w_ = 2000–3000 g.mol^−1^) was obtained from BASF and was used without further purification.

### 2.2. Formulation of ASD

Amorphous solid dispersions of TFD in PVP were formulated by co-milling at room temperature using a high energy planetary mill (Pulverisette 7 from Fritsch GmbH, Idar-Oberstein, Germany). One gram of a chosen amount of crystalline TFD and PVP previously dried for 3 h at 60 °C was placed in a 43 cm^3^ ZrO_2_ milling jar with seven ZrO_2_ milling balls (diameter of 15 mm), which corresponds to a ball/sample weight ratio of 75:1. Milling was performed at a rotation speed of 400 rpm by alternating 10 min milling periods with 5 min pauses (in order to limit heating) for a total milling time of 10 h. After milling, we checked by X-ray powder diffraction that only an amorphous halo was detected, thereby confirming that an amorphous dispersion of TFD in PVP has been obtained. Five ASDs were formulated with different TFD weight fractions: 10 wt.%, 30 wt.%, 49 wt.%, 70 wt.%, and 90 wt.%.

### 2.3. Temperature Modulated Differential Scanning Calorimetry (MDSC)

Temperature modulated differential scanning calorimetry (MDSC) experiments were carried out using a DSC Q200 from TA Instruments (Guyancourt, France) equipped with a refrigerated cooling system. Samples (m < 5 mg) were placed in a T_0_ aluminium pan (no lid) and an empty pan was used as a reference. Measurements were conducted under nitrogen atmosphere with a flow rate of 50 mL.min^−1^ applying a heating rate of 5 °C.min^−1^ with a temperature modulation of ± 0.531 °C per 40 s. Temperature and enthalpy calibrations were performed with high purity indium and *Cp* calibration for MDSC measurements was performed with sapphire.

### 2.4. Solubility of Terfenadine into PVP Matrices

The solubility limit of TFD in PVP has been established by the method developed by Latreche and co-workers [19]. The ASD obtained by co-milling 70 wt.% TFD with 30 wt.% PVP was stored for 3 h in a desiccator under a saturated ethanol atmosphere in order to induce partial re-crystallisation of small TFD crystallites finely dispersed within an amorphous matrix of TFD and PVP. This sample was placed in the DSC oven, heated at 5 °C.min^−1^ up to 100 °C and maintained at this temperature for 1 h to remove all traces of sorbed solvent. The dissolution of the TFD crystallites was then investigated: The sample was heated at 5 °C.min^−1^ to *T_a_* = 120 °C and annealed at this temperature for 5 h in order for TFD crystallites dissolve until the equilibrium state was reached. The glass transition temperature, *T_g_*, of the remaining amorphous blend was measured, after a quench to room temperature, during a subsequent heating at 5 °C.min^−1^ ± 0.531 °C per 40 s. The report of this value on the Gordon–Taylor diagram representing the evolution of *T_g_* versus the TFD content allows to deduce the equilibrium TFD concentration achieved at *T_a_*. Successive cycles of annealing at *T_a_*/quench/heating, with *T_a_* varying from 120 °C to 140 °C by steps of 5 °C, have been applied to determine the solubility limit at these annealing temperatures. It is worth noting that the annealing time of 5 h has been previously optimised by measuring, at the lowest annealing temperature (*T_a_* = 120 °C), the minimum time required to no longer observe an endotherm of dissolution on annealing and an evolution of the glass transition on the subsequent heating.

### 2.5. Dielectric Relaxation Spectroscopy (DRS)

Dielectric relaxation spectroscopy experiments were carried out using a Novocontrol Technologies GmbH (Montabaur, Germany) Alpha Analyzer equipped with a Quatro Cryosystem to control the temperature. A small amount of milled samples was put in between two gold-coated electrodes (diameter of 20 mm) and inert silica spacers (50 µm thickness) were added to avoid a short circuit between electrodes. The powder was packed as much as possible to fill the volume between the electrodes. Before recordings, samples were annealed for 2 h at 80 °C in order to remove the small amount of adsorbed water. Indeed, because of the hygroscopicity of PVP, on one hand, and the milling process that increases the reactivity by increasing the surface area, on the other hand, samples formulated by co-milling are sensitive to water. Samples were submitted to a sinewave electrical field with frequency, *F*, varying from 10^6^ Hz to 0.1 Hz, and the complex permittivity ε*(*F*) = *ε′*(*F*) − *i ε″*(*F*) where *ε′*(*F*) and *ε″*(*F*) are, respectively, the real and imaginary parts of ε*(*F*) and *i* is the imaginary number, was recorded during isotherms ranging from 80 °C to 170 °C. It should be noted that, for samples initially in the form of powder, the softening and coalescence of grains when heated can cause a variation in the capacitance that induces a variation in ε*(*F*). The frequency at which relaxation modes appear is not influenced but the magnitude of the signal is then biased. Therefore, a second series of measurements was recorded and analysed after checking that the frequencies at which relaxations appear and their shape were similar to those in the first series of recordings.

In the recorded spectra, the main relaxation was often partially hidden by ohmic conductivity and electrode polarisation developing at high temperatures and low frequencies. In order to properly analyse the relaxation, the method proposed by Wübbenhorst and Van Turnhout was used [20]. It is based on the calculation of *ε″* from the angular frequency *ω* logarithm first derivative of the real part, *ε′*, of the complex permittivity:

(1) $$\varepsilon_{der}^{\prime \prime }=-\frac{\pi }{2}\frac{\partial \varepsilon^{\prime }\left(\omega \right)}{\partial \mathrm{Ln}\omega }$$

For relaxation peaks large enough, such as those of the *α* relaxation or secondary relaxation, *ε″_der_* roughly corresponds to *ε″* without ohmic conductivity. The use of this method permits to enhance the resolution of convoluted relaxation modes and to mathematically suppress the ohmic conductivity [20].

For each isothermal evolution of *ε″_der_* with the frequency of the field, the relaxation time characteristic of the main relaxation was determined from the frequency, *F_max_*, of the peak maximum: *τ_max_*(*T*) = 1/(2π *F_max_*). When the main relaxation showed two components, two Havriliak–Negami (HN) functions [21] were used in order to deconvolute both components:

(2) $$\varepsilon^{*}\left(\omega \right)=\varepsilon_{\infty }+\frac{\Delta \varepsilon }{{\left[1+{\left(i\omega \tau_{HN}\right)}^{\alpha_{HN}}\right]}^{\beta_{HN}}}$$

where Δ*ε* = *ε_s_* − *ε_∞_* is the difference between the real permittivity values at, respectively, low and high frequency limits, *τ_HN_* is the Havriliak–Negami characteristic relaxation time of the process, and *α_HN_* and *β_HN_* are shape parameters accounting for the broadening and asymmetry of the relaxation (0 < *α_HN_* ≤ 1, 0 < *β_HN_* ≤ 1). From the HN fitting, the relaxation time associated with the maximum of each function, *τ_max_*, was then determined.

## 3. Results

### 3.1. Phase and State Diagram of TFD/PVP Blends

**Figure 1:**
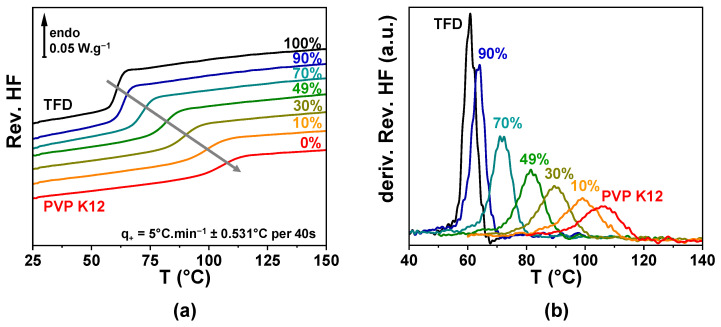
(**a**) Reversible heat flows of ASDs with 10 to 90 wt.% TFD recorded by MDSC at 5 °C.min^−1^ ± 0.531 °C per 40 s. Thermograms of pure PVP and TFD are also reported. (**b**) Temperature derivatives of signals reported in (**a**).

ASDs of TFD and PVP containing 10 wt.%, 30 wt.%, 49 wt.%, 70 wt.%, and 90 wt.% TFD were prepared by a 10 h co-milling at room temperature (see Section 2.2). In order to study the homogeneity and the stability of these ASDs, samples were analysed by temperature modulated differential scanning calorimetry (MDSC). A first heating to 180 °C at 5 °C.min^−1^ ± 0.531 °C per 40 s was recorded. However, the glass transition temperature range proved challenging to analyse as different phenomena occur at the same time, inherent to the milled nature of the samples: On one hand, a small loss of adsorbed water appears between room temperature and 75 °C and, on the other hand, a poor thermal conductivity can create some artefacts on thermograms when it is modified by the softening and spreading of powder grains. The analysis of the glass transition temperature range was, thus, realised on a second heating of the sample (after a quench down to room temperature). The reversible heat flows recorded for the different ASDs are reported in Figure 1a and the corresponding temperature derivatives in Figure 1b. Whatever the TFD content, only a single *Cp*-jump characteristic of the glass transition can be observed (single peak on the derivative signals), which indicates that TFD and PVP are mixed at the molecular level in all the concentration range. A single glass transition is usually considered as the mark of a homogeneous mixing at the molecular scale of the compounds. This *Cp*-jump shifts towards higher temperatures as the fraction of PVP in the blend increases, while its magnitude decreases and its width increases. Therefore, the features of the glass transition of the ASD shift from the ones of amorphous TFD towards the ones of PVP with the composition of the blend (see Table 1).

**Figure 2:**
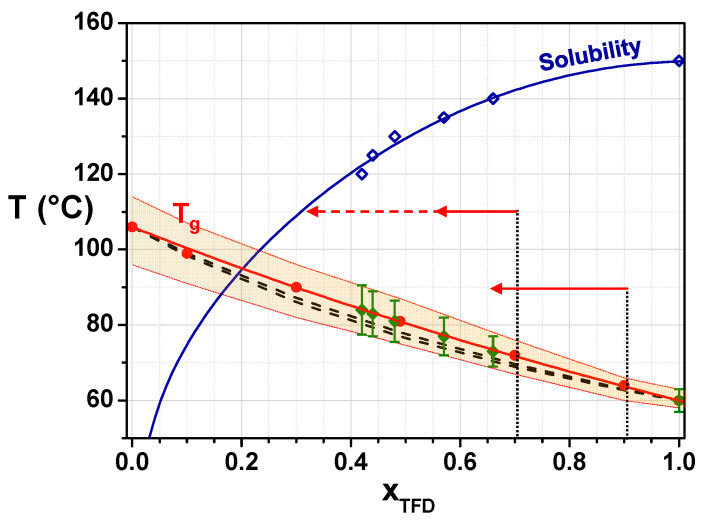
Evolution versus the TFD weight fraction, *x_TFD_*, of the *T_g_* of TFD/PVP ASDs (Gordon–Taylor diagram): Filled circles represent *T_g, midpoint_* and the orange area delimits the *Cp*-jump. *T_g_* evolution has been fitted by a Gordon–Taylor law (red solid line); the dashed lines represent the evolutions predicted in the case of a regular solution (with *K_CK min_* and *K_CK max_*, see text). The solubility limit of TFD in PVP is also reported: Experimental points (open diamonds) have been determined from the *T_g_* values (filled diamonds) of the remaining amorphous blends (see text). The evolution of the solubility versus *x_TFD_* has been fitted by a Flory–Huggins law (blue solid line). Arrows indicate the evolution of the ASD TFD content observed by DRS during isotherms at 90 °C and 110 °C (see text).

The evolution of the glass transition temperature, *T_g, midpoint_*, of the ASD with the TFD weight fraction, *x_TFD_*, is reported in Figure 2 (filled circles). It can be fitted using the Gordon–Taylor equation [22]:

(3) $$T_{g}(x_{TFD})=\frac{x_{TFD}T_{g,TFD}+K_{GT}\left(1-x_{TFD}\right)T_{g,PVP}}{x_{TFD}+K_{GT}{(1-x}_{TFD})}$$

where *T_g,TFD_* and *T_g,PVP_* are the *T_g, midpoint_* values of, respectively, pure TFD and PVP K12, *x_TFD_* is the weight fraction of TFD, and *K_GT_* is a fitting parameter. The best fit is obtained with *K_GT_* = 0.76 ± 0.02 and is reported in Figure 2 (red solid line). In the framework of the Couchman–Karasz formalism [23], the fitting parameter should be equal, in the case of a regular solution, to the ratio of the magnitudes of the *Cp*-jump associated with the glass transition of pure PVP and TFD:

(4) $$K_{CK}=\frac{\Delta {Cp}_{PVP}}{\Delta {Cp}_{TFD}}$$

With *ΔCp_PVP_* = 0.34 ± 0.02 J.g^−1^.°C^−1^ and *ΔCp_TFD_* = 0.56 ± 0.01 J.g^−1^.°C^−1^, the value *K_CK_* = 0.61 ± 0.05 is found. It is slightly lower than the value of *K_GT_*, which results in ASD’s *T_g_* values predicted in the case of an athermal solution being slightly lower than the experimental ones (see *T_g, pred_* values in Table 1 and dashed lines in Figure 2 corresponding to *K_CK min_* = 0.56 and *K_CK max_* = 0.66). This small deviation indicates the development of weak attractive interactions between TFD and PVP molecules [6].

As far as the thermal stability of the ASD produced by co-milling is concerned, only the thermogram (recorded on the first heating up to 180 °C) of the blend containing 90 wt.% TFD showed an exotherm of re-crystallisation (between 110 °C and 137 °C) followed by the endotherm of dissolution of re-crystallised TFD into the PVP matrix at higher temperatures. Since TFD amorphised by milling, contrary to the glass obtained by quench from the melt, re-crystallises on heating due to residual nuclei left by milling [17], it indicates that the PVP matrix allows to prevent the re-crystallisation of TFD (or more precisely the growth of nuclei) on heating at 5 °C.min^−1^ up to 70 wt.% TFD in the blend. One can add that, when the ASD containing 90 wt.% TFD is annealed at 90 °C for 8 h, the thermogram recorded on the isotherm shows an exotherm of re-crystallisation extending over 250 min, whereas, in the same conditions, complete re-crystallisation of TFD amorphised by a 20 h milling is achieved in 50 min (see Figure S1). Only 10 wt.% PVP is, thus, sufficient to multiply by five the time needed for re-crystallisation. Moreover, the comparison of the enthalpy of crystallisation measured for the ASD, *ΔH_cr_* = 58 ± 1 J.g^−1^ of TFD (*ΔH_cr_* = 52 ± 1 J.g^−1^ of ASD), with the one of milled TFD, *ΔH_cr_* = 76 ± 1 J.g^−1^, reveals that only 76 wt.% of the TFD contained in the ASD re-crystallises. It then remains a stable amorphous blend that contains 68 wt.% TFD. This result suggests that co-milling 90 wt.% TFD and 10 wt.% PVP leads to the formation of an ASD supersaturated with TFD. On heating, the excess TFD re-crystallises until the limit of solubility is reached.

**Figure 3:**
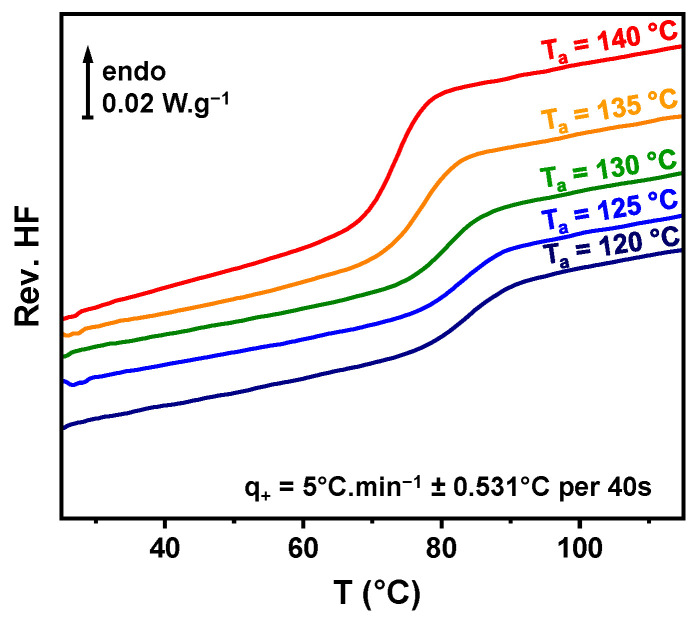
Reversible heat flows recorded by MDSC at 5 °C.min^−1^ ± 0.531 °C per 40 s of the ASD with 70 wt.% TFD after a 5 h annealing at T_a_.

In order to establish the evolution of the solubility of TFD into PVP with increasing temperatures, the method developed by Latreche and co-workers [19] has been used (see Section 2.4). Figure 3 presents the thermograms (reversible heat flows) of blends saturated with TFD obtained after 5 h of annealing at *T_a_* values ranging from 120 °C to 140 °C in 5 °C increments. A shift in the glass transition to lower temperatures is observed with increasing *T_a_*, indicating the enrichment of TFD of the remaining amorphous phase. The glass transition temperatures and the corresponding reached TFD saturation concentrations are reported in Figure 2 (respectively, filled and open diamonds) and in Table 2. It should be noted that below *T_a_* = 120 °C, the dissolution is so slow that equilibrium cannot be reached in a reasonable time.

Despite the few available points, a tentative fit of the solubility limit by the Flory–Huggins model [24] has been performed:

(5) $$\left(\frac{∆H_{m}}{R}\right)\left(\frac{1}{T_{m}}-\frac{1}{T}\right)=\ln\phi_{TFD}+\left(1-\frac{1}{\lambda }\right)\left(1-\phi_{TFD}\right)+\chi {\left(1-\phi_{TFD}\right)}^{2}$$

where *T_m_* and Δ*H_m_* are, respectively, the melting temperature and enthalpy of TFD (*T_m_* = 150 ± 1 °C, Δ*H_m_* = 54.2 ± 0.5 kJ.mol^−1^), *R* is the gas constant (*R* = 8.314 J.mol^−1^), *ϕ_TFD_* is the volume fraction of TFD, *λ* is the molar volume ratio of PVP to TFD, and *χ* is the Flory–Huggins parameter describing the interactions between the two compounds. *ϕ_TFD_* is calculated from the densities of TFD and PVP K12 (*ρ_TFD_* = 1.05 g.cm^−3^ obtained by molecular dynamics simulations and *ρ_PVP_* = 1.17 g.cm^−3^ from Matsumoto et al. [25]):

(6) $$\phi_{TFD}=\frac{\frac{x_{TFD}}{\rho_{TFD}}}{\frac{x_{TFD}}{\rho_{TFD}}+\frac{1-x_{TFD}}{\rho_{PVP}}}$$

*λ* is calculated from the densities of TFD and PVP K12 and their molar masses, *M_TFD_* = 471.7 g.mol^−1^ and *M_w PVP_* = 2500 g.mol^−1^:

(7) $$\lambda =\frac{M_{w\;PVP}\cdot \rho_{TFD}}{\rho_{PVP}\cdot M_{TFD}}$$

The value *χ* = −2.3 ± 0.1 has been obtained for the Flory–Huggins parameter. The negative value suggests the development of attractive interactions between TFD and PVP K12. For the system Indomethacin/PVP K12, Sun et al. [26] and Mahieu et al. [27] have respectively reported the values *χ* = −8.2 ± 0.6 and *χ* = −8.6 ± 0.7. For Nifedidine/PVP K12, Sun et al. [26] reported the value *χ* = −2.5 ± 0.2 and established that interactions between Indomethacin and PVP were stronger (H-bonds) than the ones developing between Nifedipine and PVP K12. In the system Curcumin/PVP K12, the value *χ* = −5.54 ± 0.15 [28] was reported and associated with quite strong interactions. The value found for TFD/PVP K12 is quite close to the one found for Nifedipine/PVP K12. This result confirms that weak attractive interactions develop between TFD and PVP in agreement with the evolution of the ASD’s *T_g_* with the TFD content.

The combination, in Figure 2, of the phase diagram (solubility curve of TFD in PVP) and the state diagram (*T_g_* evolution with TFD content) of the TFD/PVP K12 system allows to better comprehend the physical state of this system and its evolution with temperature and time. In Figure 2, above the solubility curve, the PVP matrix is undersaturated with TFD: The blend is homogeneous (one single phase) and amorphous, and it remains so. Below the solubility curve, the PVP matrix is supersaturated with TFD and the TFD in excess tends to re-crystallise. However, the kinetics depend on the molecular mobility: Below *T_g_*, its slowing down is such that the re-crystallisation is extremely slow or even blocked. Extrapolation of the solubility curve to room temperature shows that all ASDs formed by co-milling are supersaturated with TFD. Since the milling is performed at room temperature, so almost 40 °C below the glass transition of TFD, it is the milled-induced amorphisation of TFD that occurs at the same time as the mixing with PVP that permits reaching such supersaturated states. When ASDs are heated above *T_g_*, the release of the molecular mobility allows the re-crystallisation of the TFD in excess. However, due to the relatively slow kinetics of demixing/re-crystallisation, re-crystallisation is only observed on heating at 5 °C·min^−1^ for the ASD containing 90 wt.% TFD, which is the ASD that has the lowest *T_g_* and the largest gap between *T_g_* and the temperature at which the solubility limit is reached. When the system is given more time, for instance, during the annealing at 90 °C, the TFD in excess re-crystallises and the TFD content remaining in the amorphous blend tends towards the equilibrium saturated concentration. It is interesting to note, however, that according to Figure 2, this content should be close to 20 wt.%, whereas experimentally, after 8 h, 68 wt.% TFD still remains in the amorphous blend. As TFD re-crystallises and the TFD content remaining in the blend decreases, the temperature difference from *T_g_* narrows and the molecular mobility is slowed down. This limitation of mobility certainly explains why the demixing/re-crystallisation seems blocked.

### 3.2. Main Dynamics of TFD/PVP ASDs

**Figure 4:**
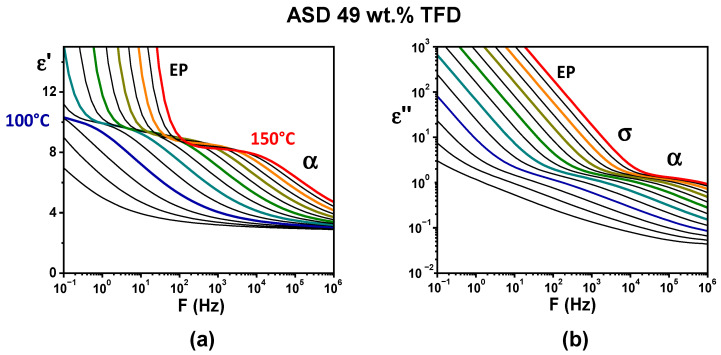
DRS spectra recorded for the ASD with 49 wt.% TFD on isotherms ranging from 85 °C to 150 °C in 5 °C increments when the frequency of the applied electrical field varies from 1 MHz to 0.1 Hz (colours help to distinguish spectra for increasing temperatures, from blue at 100°C to red at 150°C): (**a**) recordings of the real part of the complex permittivity; (**b**) recordings of the imaginary part of the complex permittivity.

**Figure 5:**
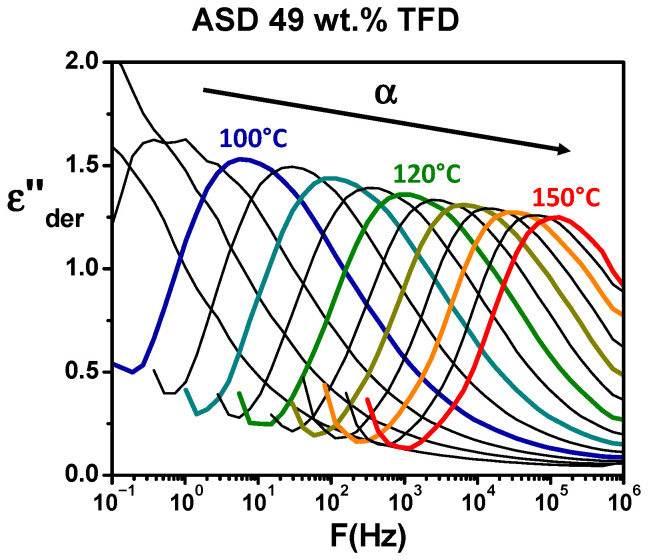
Evolution of *ε″_de_*_r_ for the ASD with 49 wt.% TFD on isotherms ranging from 90 °C to 150 °C in 5 °C increments calculated from the derivative of *ε′* values reported in Figure 4a (see text). For sake of clarity, the response due to the electrode polarisation appearing at low frequency has been cut.

In order to investigate the dynamics of ASDs in the supercooled liquid, dielectric relaxation spectroscopy (DRS) experiments have been performed (see Section 2.5). Figure 4 shows the evolution, versus the frequency of the applied electrical field, of the complex permittivity of the ASD containing 49 wt.% TFD recorded for isotherms ranging from 85 °C to 150 °C (real part, *ε′*, in Figure 4a and imaginary part, *ε″*, in Figure 4b). In Figure 4a, a step-like decrease in *ε′* shifting towards high frequencies for increasing temperature can be seen, which suggests the shift of a relaxation mode. However, a clear observation of a peak on *ε″* is prevented by the conductivity (σ) and the electrode polarisation (EP) appearing on lower frequencies. In order to obtain the ohmic-conduction free dielectric loss, *ε″* was calculated from the logarithmic derivative of the real part (denoted *ε″_der_*, see Section 2.5). The obtained spectra are reported in Figure 5. Now, the shift towards high frequency with increasing temperature of a peak is clearly visible, revealing the *α* (or main) relaxation associated with the dynamic glass transition.

**Figure 6:**
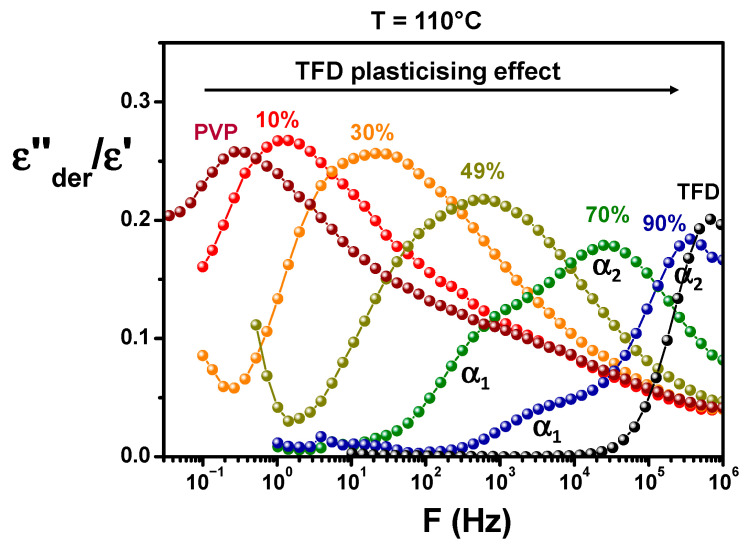
Comparison at 110 °C of the evolution versus the frequency of the field of the ratios *ε″_de_*_r_/*ε′* for ASDs with different TFD weight fractions. Pure PVP and TFD are also reported.

The same type of experiments and analyses have been performed for the ASDs of different TFD contents. Recorded spectra can be seen in the Supplementary Materials (see Figure S2). It should be pointed out that, in the case of the ASD containing 90 wt.% TFD, re-crystallisation occurs on heating as observed by DSC. Therefore, measurements presented were recorded during a second series of isotherms (after melting), but we checked that the low-temperature recordings were identical to those obtained during the first series before re-crystallisation. Figure 6 shows the comparison at 110 °C of the ASDs and pure compounds responses. It should be noted that to avoid a difference in the magnitude of spectra induced by a filling of the capacitor formed by the sample and the electrodes that could differ from one sample to another (samples being initially in the form of powder), the values of *ε″_der_* calculated from *ε′* have been normalised by *ε′* values. From the analysis of Figure 6, it appears, firstly, that, when the content of PVP in the blend increases, the main relaxation appears at lower frequencies, which means the dynamics is slowed down. It clearly shows the anti-plasticising effect of PVP on TFD/PVP blends already observed by MDSC. Secondly, the magnitude of the *α* relaxation tends to decrease as the TFD content increases, in agreement with the relative magnitudes of pure compounds relaxation. Lastly, one can observe a change in the shape of the relaxation mode with the ASD composition. For a TFD content of 10 wt.%, the shape of the mode closely resembles that of pure PVP and is mainly marked by a broadening of the high frequency flank of the peak above 500 Hz corresponding to the overlapping with a secondary relaxation mode of PVP. This broadening can also be observed, to a lesser extent, for the ASD containing 30 wt.% TFD. However, independently of this effect, it can be seen that, when the TFD content increases (the PVP content decreases), the mode broadens (particularly visible for 49 wt.% TFD) and splits into two components with the appearance of an increasingly pronounced shoulder on the low-frequency side (70 wt.% and 90 wt.% TFD). It should be underlined that this shoulder is not an artefact resulting from the mathematical treatment of data. Indeed, for ASD containing 90 wt.% TFD, a slight shoulder can be detected (see Figure S3a) on the low-frequency side of the relaxation mode on the *ε″*(*F*) spectra recorded at the highest temperatures where the conductivity and electrode polarisation are more decoupled from the main relaxation (for example, at 100 °C) and it appears in the same frequency range as the low-frequency component observed on *ε″_der_*(*F*) (~10^4^ Hz at 110 °C). Therefore, although hardly visible on *ε″*(*F*) recordings due to partial overlap with the conductivity phenomenon, this component exists and is clearly revealed when the conductivity is suppressed by the mathematical treatment yielding *ε″_der_*(*F*). Moreover, the analysis of the complex dielectric modulus M*(*ω*) = 1/ε*(*ω*) shows that the shoulder can also be detected on the evolution versus frequency of the imaginary part *M″* not sensitive to electrode polarisation: It appears on the low-frequency side of the mode on recordings at the highest temperatures where the conductivity peak is more decoupled from the main relaxation (see Figure S3c). Therefore, the shoulder observed at low frequencies on the *ε″_der_*(*F*) spectra for the ASDs with a high TFD content (70 wt.%, 90 wt.% TFD) is a genuine relaxation mode and not an artefact resulting from the mathematical treatment of data or from the flow of free charge carriers.

**Figure 7:**
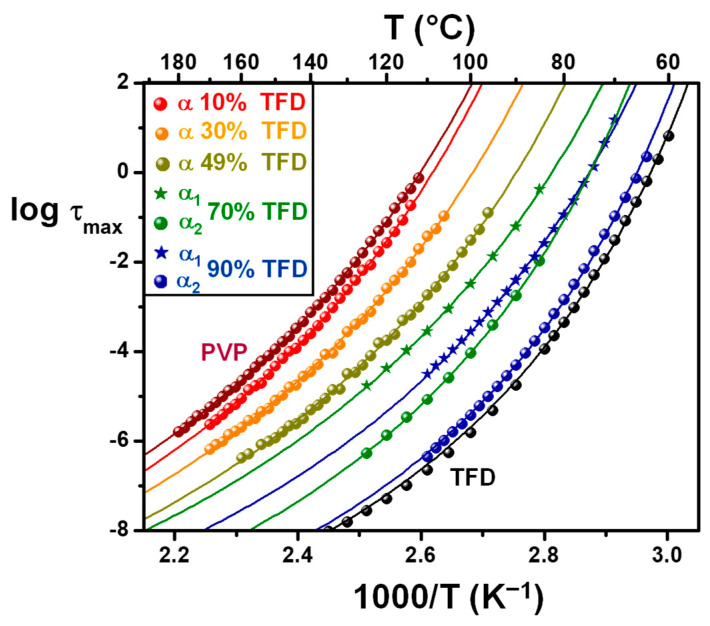
Arrhenius diagram of the temperature evolution of the relaxation times *τ_max_* associated with the *α* mode or the *α_1_* and *α_2_* components for the ASDs with different TFD weight fractions. The evolutions of *τ_max_* in the case of pure PVP and TFD [17] are also reported. Solid lines represent fits by VTFH laws (see text).

The relaxation time associated with this main relaxation has been determined from the frequency of the peak maximum for pure PVP and ASDs containing 10 wt.%, 30 wt.%, and 49 wt.% TFD. In this latter case, although the broadening of the mode suggests the dissociation into two components, they are too close to one another to be deconvoluted. For ASDs containing 70 wt.% and 90 wt.% TFD, *ε″_der_*(*F*) spectra were fitted with two HN functions (see Section 2.5) allowing to extract the value of the relaxation time associated with the *α_1_* and *α_2_* modes corresponding, respectively, to the low- and high-frequency components. The temperature evolutions of these relaxation times are reported in an Arrhenius diagram in Figure 7, along with the evolution of the relaxation time of the *α* mode of pure PVP and pure TFD previously published in [17]. These evolutions are clearly non-linear, and they have been fitted by a Vogel–Tammann–Fulcher–Hesse (VTFH) law [29,30,31]:

(8) $$\log\tau =\log\tau_{\infty }+\frac{B}{\ln\left(10\right)\;\left(T-T_{0}\right)}$$

where *τ_∞_* would be the relaxation time at infinite temperature, *T*_0_ is the so-called Vogel temperature at which the relaxation time would diverge, and *B* is a parameter that accounts for the curvature of the evolution. Owing to the lack of points for some blends, especially at low frequencies, and in order to compare the behaviour laws, the value of log *τ_∞_* has been fixed to −14 for all evolutions. The obtained values of *B* and *T*_0_ are reported in Table 3.

Usually, the temperature at which the relaxation time characteristic of the *α* relaxation of a glass-forming liquid reaches 100 s corresponds to the glass transition temperature. The extrapolation at *τ* = 100 s of the VTFH fits of the relaxation times of the ASDs relaxation modes are reported in Table 3 (*T_g, DRS_*). The values deduced from the extrapolation of the VTFH fits of the *α* modes or the *α_2_* components are in good agreement with the *T_g_* values deduced from the MDSC experiments (see Table 1) since *T_g, DRS_* either corresponds to the *T_g, onset_* for blends with the higher TFD contents (usually the case of small molecules) or is included in the *T_g, onset_* − *T_g, midpoint_* interval for blends with the higher PVP contents (the glass transition being larger for polymers) (see Table 1 and Figure 2). The degree of the departure of the temperature evolution of the relaxation time from an Arrhenius behaviour is usually quantified by the fragility index *m*, defined as follows [32,33,34]:

(9) m=dlog⁡τmaxTdTgTT=Tg=B Tgln10 Tg−T02

The fragility index can vary between 16, when there is no deviation from the Arrhenius law (liquids classified as strong [35]) and 170, for the most important deviations [36] (liquids classified as fragile [35]). The calculated fragility indexes are reported in Table 3: As the TFD content increases, they vary between the two extreme values *m* = 70 (moderately fragile) and *m* = 114 (very fragile) for, respectively, pure PVP and TFD. Since the value of the pre-exponential factor *τ_∞_* was fixed at 10^−14^ s, the evolution of *m* with the TFD content is directly linked to the difference *T_g, DRS_* − *T_0_* (approximately equal to the ratio *T_g_*/*m*) according to Angell [37].

The temperature evolution of the relaxation time of the *α_1_* component of ASDs containing 70 wt.% and 90 wt.% is well described by a VTFH law and the obtained values of the fitting parameters are well in line with those of *α* modes or *α_2_* components, which supports well that this relaxation mode can be attributed to a dynamic glass transition. The findings for ASDs with 70 wt.% and 90 wt.% TFD of two modes, *α*_1_ and *α*_2_, associated with wide amplitude motions points out a heterogeneity of the main dynamics. This heterogeneity of the dynamics suggests a structural heterogeneity of ASDs. It can indicate that, for TFD content higher than 70 wt.%, the TFD/PVP blends are heterogeneous and consist of two amorphous phases of different compositions, each having its own main dynamics (*α*_1_ and *α*_2_) characterised by a relaxation time. It is challenging to determine the TFD content of each phase, but an estimate can be made by comparing the evolution of the relaxation times associated with *α*_1_ and *α*_2_ modes. For example, for the ASD with 90 wt.% TFD, one can observe in Figure 7 that the *α*_1_ mode is very close to the *α*_2_ mode of the ASD with 70 wt.% TFD. This suggests that, in this blend, two amorphous phases coexist: one (*α*_2_) containing around 90–95 wt.% TFD and another (*α*_1_) containing slightly less than 70 wt.% TFD. It is not possible to determine the proportion of each phase in the blend but given the relative amplitudes of *α_1_* and *α_2_* modes, the former is very much in the minority. As for the ASD with 70 wt.% TFD, the comparison of the results obtained for the *α_1_* with the other *α* or *α*_2_ modes suggests that one phase (*α*_2_) contains around 70 wt.% TFD while the other (*α*_1_) contains around 60 wt.% TFD, the proportion of the latter although in the minority being quite important.

The striking point is that the thermodynamic investigation of the ASDs by MDSC did not reveal any heterogeneity since a single *Cp*-jump was observed whatever the TFD content. Furthermore, no sign of a second glass transition was detected on the signal derivative (see Figure 1). In addition, the application of a specific MDSC protocol allowing a dynamic study (heat capacity spectroscopy) revealed no heterogeneity of the dynamics, in particular for the ASDs with 70 wt.% and 90 wt.% TFD. However, previous studies have already reported the lack of reliability of a distinctive single glass transition as an indicator of the homogeneity of ASDs [38]. Here, DRS appears as a technique more sensitive to structural heterogeneities than the thermodynamic techniques. Since the two *T_g, DRS_* deduced from the extrapolation at *τ* = 100 s of the *α_1_* and *α_2_* components are included in the temperature range where the *Cp*-jump develops, one explanation could be that this *Cp*-jump spreads over a too-limited temperature range to allow a deconvolution of the two glass transitions. Another explanation could be that the dielectric probe (dipolar moments) is more sensitive to structural heterogeneities than the thermodynamic probe (heat flow exchanges) allowing to reveal them. These results emphasise the need of techniques other than the conventional DSC to assess the homogeneity of ASDs.

### 3.3. Mobility, Stability, and Solubility Relationships

**Figure 8:**
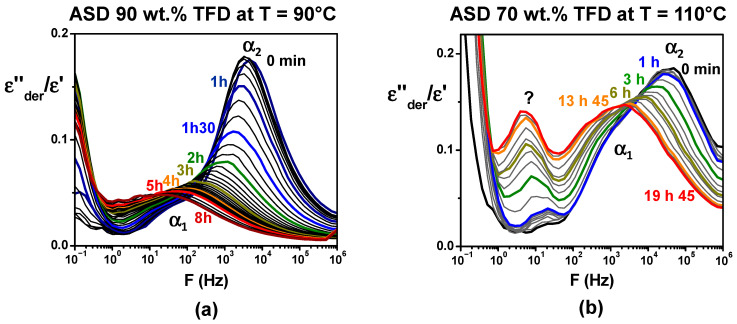
*ε″_de_*_r_/*ε′* (F) evolutions recorded over time at fixed temperature: (**a**) at 90 °C for the ASD with 90 wt.% TFD, with recordings every 10 min for 5 h then every hour until 8 h; (**b**) at 110 °C for the ASD with 70 wt.% TFD, with recordings every 10 min for 1 h then every hour until 6 h, after 7 h 45 min, and every 3 h until 19 h 45 min.

As previously mentioned, re-crystallisation of the ASD containing 90 wt.% TFD was observed during heating on a first series of recordings by DRS. We took the opportunity of such behaviour to gain information on the impact of the re-crystallisation on the main dynamics by following, for that blend, the evolution over time of the dielectric permittivity on successive recordings at 90 °C. The corresponding *ε″_der_*/*ε′* ratios are reported versus frequency in Figure 8a. It should be noted that this ratio is reported, rather than *ε″_der_*, since it suppresses amplitude variations that could be induced by the softening of powder grains during the annealing. A sharp decrease in the *α_2_* component magnitude is quickly observed, indicating a re-crystallisation of TFD, while the maximum of the mode simultaneously shifts towards low frequency as the TFD content of the corresponding remaining amorphous phase decreases. The *α_1_* mode is less affected, resulting in the merging of both components, which indicates a homogenisation of the TFD content within the amorphous blend. After 2 h, the decrease in amplitude continues, albeit less significantly, and the continuous shift towards low frequency is mainly observed. After 8 h, it leads to a broad relaxation mode appearing on *ε″_der_*(F) at 14 Hz, corresponding to log *τ* = −1.94. The normalisation by *ε′* of *ε″_der_* induces a shift in relaxations towards higher frequencies as the normalisation by *ε′* of *ε″* (tan *δ*) does. By plotting this value in the Arrhenius diagram (T = 90 °C) of Figure 7, we can roughly estimate that the remaining amorphous phase contains around 65 wt.% TFD. It is interesting to note that during the DSC monitoring of the re-crystallisation at 90 °C, we found that after 15 h, the remaining amorphous phase contained 68 wt.% TFD. The dielectric spectroscopy results are, therefore, consistent with those obtained by DSC: During an isotherm at 90 °C, the TFD re-crystallises until the remaining amorphous phase contains approximately 65–70 wt.% TFD. The same type of study was also carried out on the ASD containing 70 wt.% TFD. Figure 8b shows the evolution over time of *ε″_der_*/*ε′* recorded at 110 °C. As previously, the spectra evolve, though more slowly. The magnitude of the *α*_2_ component decreases (mainly over the first 6 h) and the mode shifts towards the *α*_1_ mode that evolves slower. It leads to a broad mode, which indicates the partial re-crystallisation of TFD and the depletion in TFD of the remaining amorphous phase. Another striking fact is the simultaneous appearance and development of a new peak at lower frequencies. It is, however, difficult, without a complete investigation of its temperature evolution, to assess whether it is an *α* relaxation associated with a new phase. After 20 h at 110 °C, it is difficult to distinguish the *α*_2_ and *α*_1_ components, but shoulders reminiscent of these components appear around 2.8 kHz and 270 Hz, while the additional peak appears at 5 Hz. It corresponds, respectively, to log *τ* = −3.58, log *τ* = −3.14, and log *τ* = −1.4. By plotting these values on the Arrhenius diagram in Figure 7, we can estimate that the remaining blend contains mainly between 50 wt.% and 60 wt.% TFD (with a few of another phase containing 30 wt.% TFD when considering the additional peak is an *α* relaxation). During a similar experiment performed by MDSC, the *T_g_* of the blend rose from 72 °C (content of 70 wt.% TFD) to 78 °C, which, plotted on the Gordon–Taylor diagram in Figure 2, corresponds to a TFD content of 55 wt.%, in good agreement with DRS results. Lastly, an isothermal study at 110 °C carried out by DRS and MDSC for the ASD with 49 wt.% TFD showed that in that case, there was no change over 36 h.

According to the phase diagram established in Figure 2, at 110 °C, the equilibrium concentration of TFD in PVP is estimated to be 30 wt.%; therefore, during an isotherm at 110 °C, ASDs initially containing 49 wt.% and 70 wt.% TFD should both re-crystallise until the remaining blends reach a TFD concentration of 30 wt.%. However, the DRS results show that, for the former, the TFD content does not change and for the latter, it also tends towards a concentration close to 50 wt.%. One can note, however, that, when the new peak is considered as an *α* relaxation, the associated phase actually contains 30 wt.% TFD. At 90 °C, the TFD equilibrium concentration in PVP is estimated to be 15 wt.%. However, as the TFD concentration decreases, the temperature of the glass transition of the blend increases, so it is expected that the TFD content will not fall below 45 wt.%–50 wt.% (corresponding to the temperature of the ending of the *Cp*-jump) (see Figure 2) because the mobility (diffusion) of TFD in PVP will be blocked. Here, again, the TFD content reached during an isotherm at 90 °C is higher (estimated content of 65 wt.%–70 wt.%). The driven amorphous solid dispersions obtained by co-milling, therefore, re-crystallise less than predicted by the solubility limit, despite long isotherms, and remain supersaturated for a longer time. This is particularly the case for the ASD with 50 wt.% TFD, which does not evolve at 110 °C for 36 h). This suggests that re-crystallisation (growth of nuclei left by milling) is blocked by a too-slow mobility (particularly of the PVP chains) at the temperatures tested, despite being 25–35 degrees higher than *T_g, end_*. In a study carried out on Indomethacin/PVP blends with PVP of different molar masses, it was shown by Mohapatra et al. that viscosity seems to be a key factor in the stability of ASDs [13]. Furthermore, Sahoo and co-workers recently related the ability of a polymer to prevent the crystallisation of the drug to the critical polymer concentration, C*, defined in the polymer solution theory, below which polymer chains tend to exclude one another [39]. The authors suggested that, above C*, the crystallisation of the drug could be limited by the limited gaps between polymer chains. Another explanation they invoked could be the rapid increase in the viscosity occurring for polymer concentrations exceeding C*. It would, therefore, be interesting to perform viscosity measurements of the TFD/PVP ASDs. Such an investigation of the ASD viscosity evolution could help determine whether there exists a limiting viscosity above which demixing/crystallisation is blocked.

## 4. Conclusions

In this article, we investigated amorphous solid dispersions of Terfenadine in PVP K12 obtained after a 10 h co-milling at room temperature. The thermodynamic characterisation by temperature modulated differential scanning calorimetry of theses ASDs enabled us to depict the evolution versus the TFD content of two keys parameters: the glass transition temperature of the blend and the temperature corresponding to the solubility limit of TFD in the polymeric matrix. The results systematically showed a single glass transition, which is generally the criterion of a blend homogeneity. The fittings, on one hand, of the *T_g_* evolution by a Gordon–Taylor law and, on the other hand, of the solubility by a Flory–Huggins law, both established the development of attractive but rather weak interactions between TFD and PVP. In contrast, the investigation by dielectric relaxation spectroscopy of the main dynamics of the ASDs revealed the heterogeneities of blends for relatively small fractions of PVP with the existence of two amorphous phases of different compositions, each characterised by its own main dynamics. This highlights the limitations of the conventional approach using DSC to characterise amorphous pharmaceutics and the calorimetric *T_g_* as a unique probe of molecular mobility. The need to use more refined methods to probe the dynamics, as DRS, is clearly demonstrated.

This research work allowed us to establish a link between the dynamics of TFD/PVP ASDs and their phase and state diagrams, which give us better insights into the mechanisms of the re-crystallisation of ASDs. The evolution of the solubility of TFD in PVP pointed out that designed ASDs are supersaturated with TFD at room temperature in all the concentration ranges. However, the results showed that PVP slows down the main dynamics of the blends. This limits the re-crystallisation of the drug on heating at 5 °C.min^−1^ that occurs only for a very high TFD content (90 wt.%). Additionally, in the supercooled liquid state, the isothermal monitoring by DRS of the re-crystallisation of supersaturated ASDs highlighted a re-crystallisation for the highest TFD contents (>70 wt.%), with the depletion in TFD first affecting the phases richest in TFD, characterised by the greatest mobility. However, the slowing down of the main dynamics induced by the decrease in TFD content leads to a slowing down of the re-crystallisation kinetics that is finally blocked by a too-low mobility, even at temperatures higher, by 25–30 °C, than the glass transition. It leads to ASDs that remain supersaturated with TFD for longer than expected from the analysis of the phase and state diagram. This underlines the intertwining of thermodynamic and dynamic aspects and the complexity of the mechanisms of the re-crystallisation of ASDs.

## Figures and Tables

**Table 1:** Experimental glass transition temperatures (*T_g, onset_* and *T_g, midpoint_*) and magnitudes (Δ*Cp*) of ASD containing different weight fractions of TFD, *x_TFD_*. Values of the ASD’s *T_g_* predicted in the case of an athermal solution with *K_CK_* = 0.61 ± 0.05 (*T_g, pred_*) are also reported.

*x_TFD_*	*T_g, onset_* (°C, ±1 °C)	*T_g, midpoint_* (°C, ±1 °C)	Δ*Cp* (J.g^−1^.°C^−1^)	*T_g, pred_* (°C)
0 (PVP)	96	106	0.34 ± 0.02	
0.10	91	99	0.36 ± 0.01	98.9 ± 0.5
0.30	82	90	0.40 ± 0.01	87.0 ± 0.9
0.49	75	81	0.45 ± 0.01	77.8 ± 0.9
0.70	67	72	0.51 ± 0.01	69.5 ± 0.6
0.90	60	64	0.54 ± 0.01	62.9 ± 0.2
1 (TFD)	58	60	0.56 ± 0.01	

**Table 2:** Glass transition temperature and TFD weight fraction, deduced from the Gordon–Taylor diagram, of the amorphous blend remaining after 5 h annealing at *T_a_*.

*T_a_* (°C)	*T_g, midpoint_* (°C, ±1 °C)	*x_TFD_* (±0.01)
120	84	0.42
125	83	0.44
130	81	0.48
135	77	0.57
140	73	0.66

**Table 3:** Values of *B*, *T*_0_, the temperature corresponding to *τ* = 100 s (*T_g, DRS_*), and the fragility index *m* extracted from the VTFH fitting of the temperature evolution of relaxation times obtained for the ASDs with different TFD content (*x_TFD_*).

*x_TFD_*		*B* (K)	*T*_0_ (°C)	*T_g, DRS_* (°C)	*m*
0 (PVP)		3145 ± 10	14.2 ± 0.4	99.5	70 ± 3
0.10		2950 ± 14	17.3 ± 0.5	97.5	74 ± 3
0.30		2851 ± 14	11.1 ± 0.6	88.5	75 ± 3
0.49		2657 ± 17	7.6 ± 0.6	79.5	79 ± 4
0.70	α_1_	2641 ± 21	0.6 ± 0.8	70.0	82 ± 5
	α_2_	2006 ± 10	12.7 ± 0.4	67.0	100 ± 5
0.90	α_1_	2356 ± 19	2.0 ± 0.6	66.0	85 ± 5
	α_2_	1759 ± 12	11.2 ± 0.4	59.0	111 ± 7
1 (TFD)		1729 ± 17	10 ± 1	56.5	114 ± 7

## Data Availability

The original contributions presented in this study are included in the article/Supplementary Material. Further inquiries can be directed to the corresponding author.

## Supplementary Materials

The following supporting information can be downloaded at: https://www.mdpi.com/article/10.3390/polym17091210/s1, Figure S1: (a) Comparison of DSC thermograms recorded at 90 °C showing the isothermal re-crystallisation of pure TFD amorphised by milling and of the TFD/PVP ASD with 90 wt.% TFD. (b) Evolution versus time of the crystalline fractions of TFD calculated from continuous integration of the exotherms of re-crystallisation observed in (a). Figure S2: ε″_der_ versus the frequency of the applied electrical field recorded at increasing temperatures for TFD/PVP ASDs with (a) 10 wt.% TFD (in 5°C increments), (b) 30 wt.% TFD (in 5°C increments), (c) 49 wt.% TFD (in 5°C increments), (d) 70 wt.% TFD (in 5°C increments), and (e) 90 wt.% TFD (in 2°C increments). Figure S3: For the TFD/PVP ASD with 90 wt.% TFD, isothermal recordings (every 2 °C) versus frequency of the applied electrical field of (a) the imaginary part, ε″, of the complex permittivity, (b) ε″_der_ calculated from the derivative of the real part of the complex permittivity, and (c) the imaginary part, M″, of the complex dielectric modulus.

## Abbreviations

The following abbreviations are used in the manuscript:

ASD	amorphous solid dispersion
DRS	dielectric relaxation spectroscopy
DSC	differential scanning calorimetry
EP	electrode polarisation
F	frequency (Hz) of the applied electrical field in DRS
HN	Havriliak-Negami
MDSC	temperature modulated differential scanning calorimetry
PVP	Polyvinylpyrrolidone
Rev. HF	reversible heat flow (W.g^−1^)
T_a_	annealing temperature (°C)
TFD	Terfenadine
T_g_	glass transition temperature (°C)
T_g, DRS_	temperature corresponding to a relaxation time of the main relaxation of 100 s (°C)
T_g, midpoint_	temperature of the glass transition midpoint (°C)
T_g, onset_	onset temperature of the glass transition (°C)
T_g, pred_	glass transition temperature (°C) predicted from the Couchman-Karasz model
VTFH	Vogel-Tammann-Fulcher-Hesse
x_TFD_	weight fractions of TFD
wt.%	weight percentage
