# Supplementary material for: Exploring the Relationship Between Stability and Dynamics in Polymer-Based Amorphous Solid Dispersions for Pharmaceutical Applications
Source: Polymers (Basel). 2025 Apr 28;17(9):1210. doi: 10.3390/polym17091210 (PMC12073697; doi:10.3390/polym17091210)
Supplement: Supplementary file 1 [file polymers-17-01210-s001.zip › polymers-3604193-supplementary.pdf]

Supplementary materials

Exploring the relationship between stability and dynamics in polymer-based amorphous solid dispersions for pharmaceutical applications

Emeline Dudognon*, Jeanne-Annick Bama and Frédéric Affouard

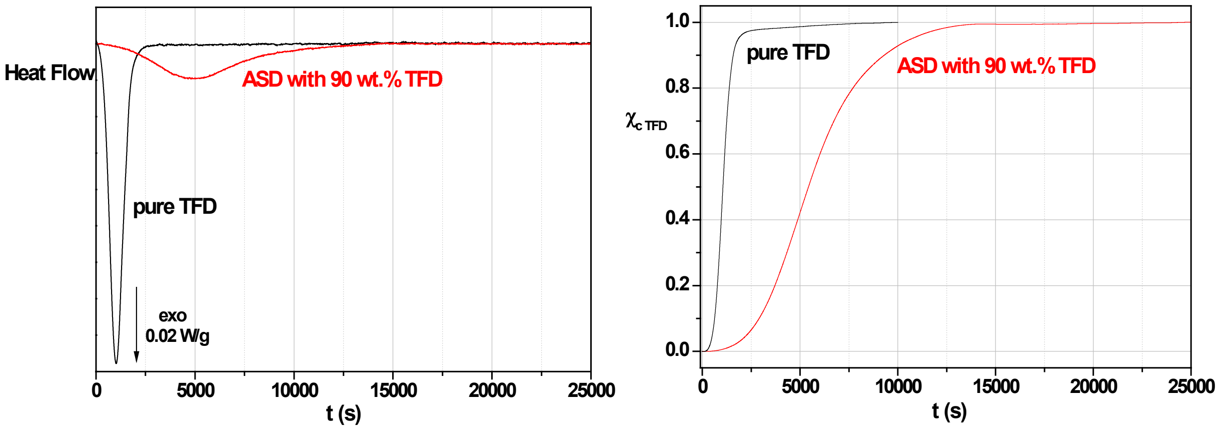

(a) (b)

Figure S1: (a) Comparison of DSC thermograms recorded at 90 °C showing the isothermal re-crystallisation of pure TFD amorphised by milling and the TFD/PVP ASD with 90 wt.% TFD; (b) evolution versus time of the crystalline fractions of TFD calculated from continuous integration of the exotherms of re-crystallisation observed in (a).

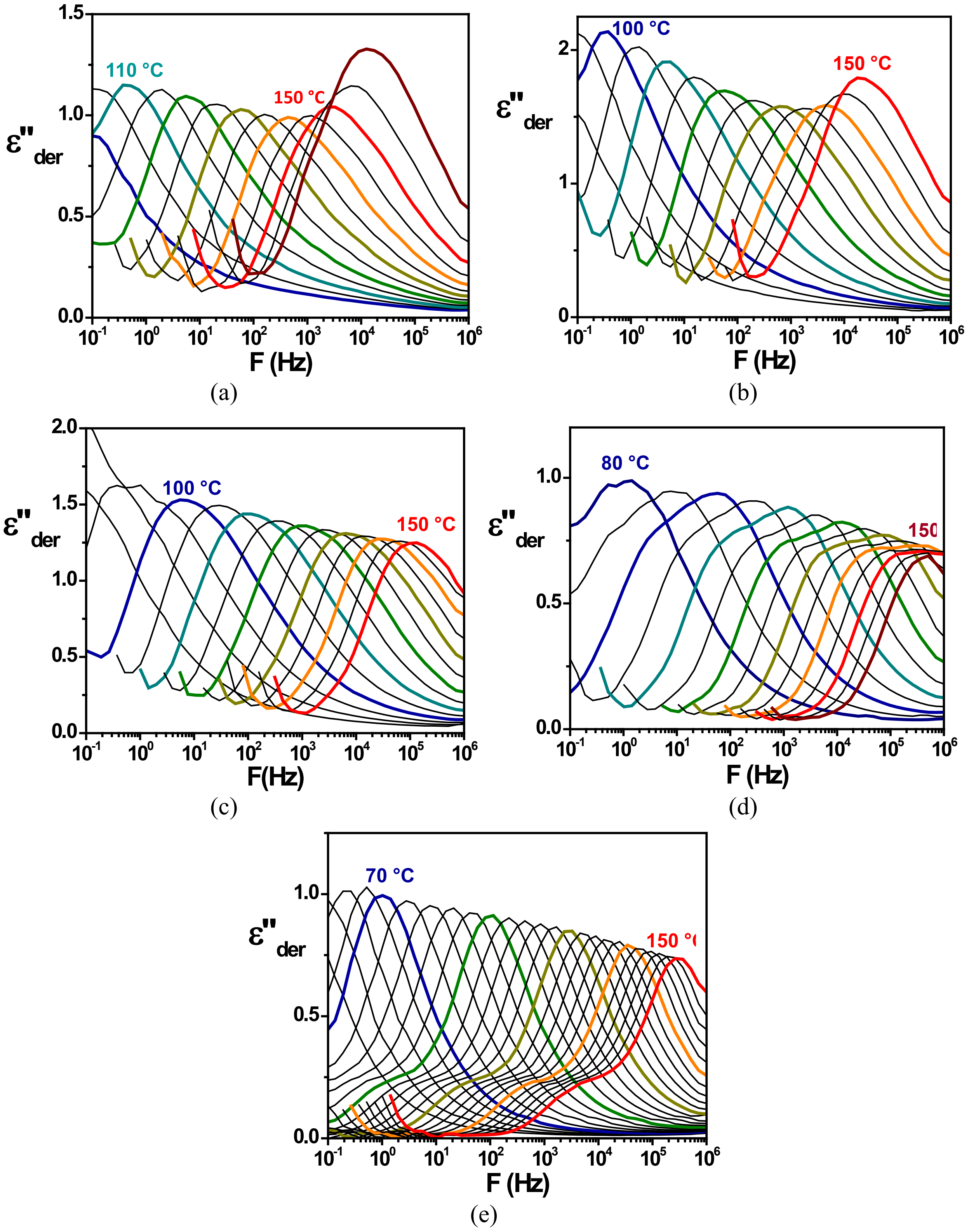

Figure S2: Evolution versus the frequency of the applied electrical field of ε''$_{der}$ recorded every 5 °C for TFD/PVP ASDs with (a) 10 wt.% TFD; (b) 30 wt.% TFD; (c) 49 wt.% TFD; (d) 70 wt.% TFD; (e) 90 wt.% TFD.

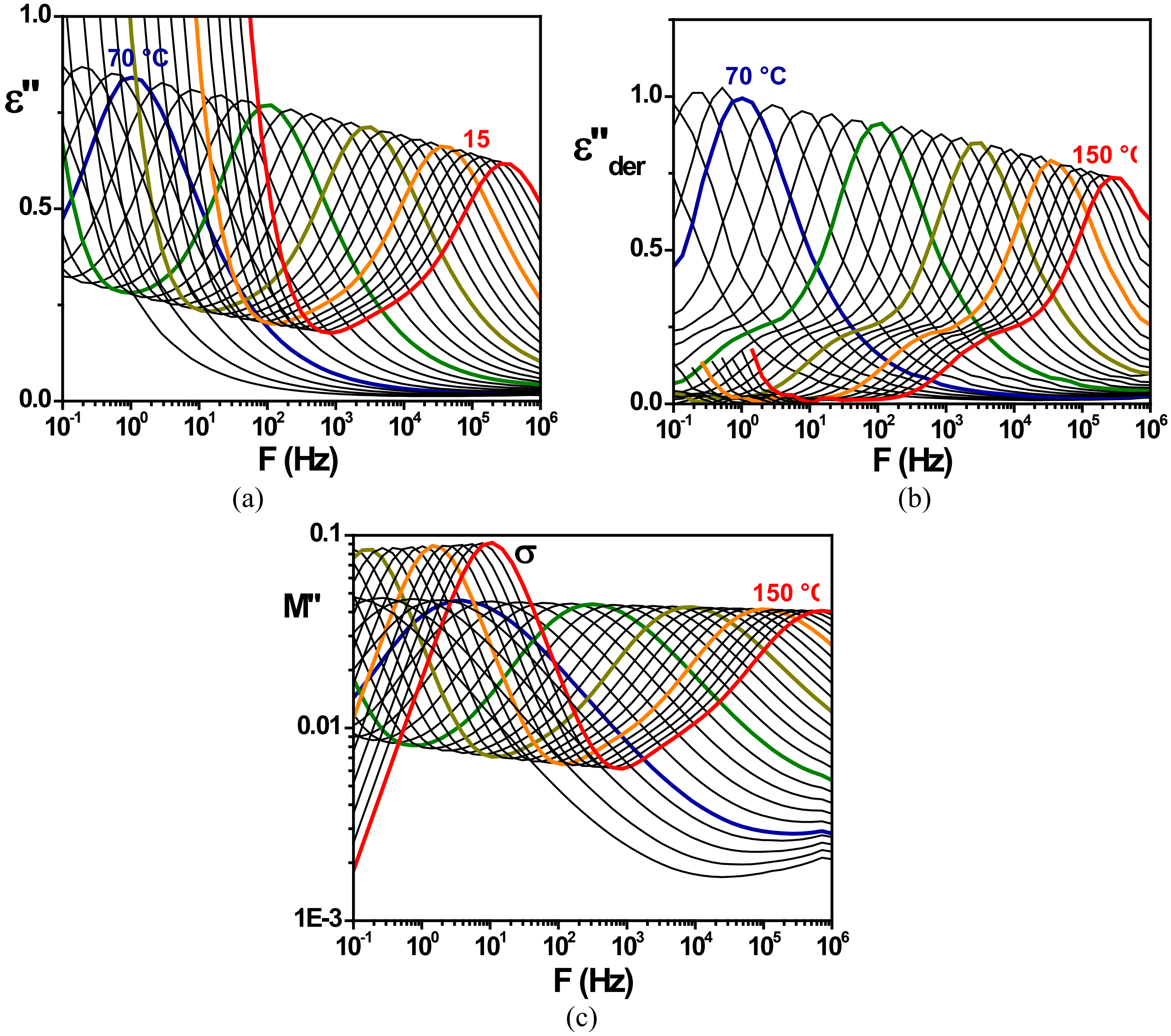

Figure S3: For the TFD/PVP ASD with 90 wt.% TFD, isothermal recordings (every 2 °C) versus the frequency of the applied electrical field of: (a) the imaginary part, ε'', of the complex permittivity; (b) ε''_{der} calculated from the derivative of the real part of the complex permittivity; (c) the imaginary part, M'', of the complex dielectric modulus.
